# 3D-driven alveolar ridge augmentation based on reverse planning: a retrospective case series study

**DOI:** 10.1186/s12903-026-09140-6

**Published:** 2026-07-04

**Authors:** Fanni Bolya-Orosz, Balint Molnar, Željka Perić Kačarević, Peter Windisch, Daniel Palkovics

**Affiliations:** 1 Department of Periodontology, Semmelweis University, Szentkirályi utca 47, Budapest, 1088 Hungary; 2 Botiss medical AG Ullsteinstraße 108, Ullsteinstraße 108, Berlin, 12109 Germany; 3 Department of Anatomy Histology and Embryology, Faculty of Dental Medicine and Health, Josip Juraj Strossmayer University of Osijek, Ul. cara Hadrijana 8/A, Osijek, 31000 Croatia

**Keywords:** CBCT, Virtual planning, Reverse engineering, Guided bone regeneration, Dense PTFE membrane, Guided surgery

## Abstract

**Background:**

Reconstruction of advanced vertical and combined alveolar ridge defects still remains a challenge in implant dentistry. Digital technologies and virtual planning may potentially improve the predictability of guided bone regeneration (GBR). This study aimed to evaluate a fully digital, reverse-planning workflow for vertical ridge augmentation using membrane-cutting guides.

**Methods:**

This retrospective case series included 15 surgical sites presenting with vertical or combined alveolar ridge defects. A digital workflow integrating cone-beam computed tomography (CBCT), intraoral scanning, and virtual prosthetic planning was used to simulate ideal implant positions and corresponding hard tissue augmentation. Membrane-cutting guides were designed and fabricated using additive manufacturing to shape dense polytetrafluoroethylene membranes. Vertical GBR was performed using a split-thickness flap design and a tent-pole approach. Linear and volumetric hard tissue changes were assessed by comparing baseline and 9-month postoperative CBCT scans.

**Results:**

Significant vertical bone gain was observed at all measurement points (*p* = 0.007), with mean increases from 15.70 mm ± 4.34 mm to 19.96 mm ± 3.83 mm at the central site. The mean volumetric hard tissue gain was 755.33 mm^3^ ± 411.22 mm^3^, closely matching the planned volume (757.50 mm^3^ ± 417.78 mm³), with no significant difference (*p* = 0.649). Using Spearman’s correlation, a strong positive correlation was found between planned and achieved volumes (Spearman’s ρ = 0.825, *p* = 0.0004). The mean augmentation efficacy was 20.13 ± 15.21 mm^3^/mm.

**Conclusions:**

The proposed 3D-driven reverse-planning workflow enabled predictable vertical ridge augmentation with high agreement between planned and achieved outcomes. This approach represents a feasible and accessible alternative to fully customized systems; however, further prospective controlled studies are required to validate these findings.

**Supplementary Information:**

The online version contains supplementary material available at https://doi.org/10.1186/s12903-026-09140-6.

## Introduction

Despite recent developments in reconstructive surgery and material sciences, reconstruction of severe vertical and combined alveolar bone atrophies still poses significant clinical challenges. However, as digital technologies are gradually integrated into reconstructive surgery, virtual surgical planning methods and navigated procedures can be used to enhance the predictability of guided bone regeneration (GBR) [1] Various patient-specific solutions, such as customized bone blocks (CABBs) [2, 3] and customized titanium meshes (CTMs) [3] produced via computer-aided design/computer-aided manufacturing (CAD/CAM), have recently been described for the reconstruction of vertical ridge deficiencies.

3D-printed CTMs have been introduced to further refine the application of titanium barriers [3, 4]. CTMs aim to avoid complications associated with prefabricated meshes (e.g., soft tissue irritation and flap dehiscence caused by sharp edges) [3]. The growing accessibility of digital approaches, including digital radiographic image processing, CAD/CAM technologies, and, more recently, artificial intelligence (AI), has made the design and fabrication of CTMs increasingly feasible. In their 2025 meta-analysis, Ragucci et al. demonstrated superior clinical usability and handling of CTMs compared to standard solutions [4]. While they found no significant difference in terms of mean vertical hard tissue gain, membrane exposure occurred more frequently in cases treated with prefabricated titanium meshes (30.9% vs. 20.3%). Other complications, such as infections, were also less frequent in the CTM group. Moreover, vertical GBR with CTMs resulted in significantly greater horizontal hard tissue gain, possibly due to better adaptation to various defect configurations and enhanced graft stabilization. With an individualized shape, important anatomical structures can be avoided during fixation, and flap adaptation might be easier, reducing the number of potential complications [4, 5].

However, various clinicians prefer block grafting over GBR due to the excellent osteoconductive, osteoinductive, and osteogenic properties of autogenous bone, as well as the reduced risk of flap dehiscence [6]. Block grafting and GBR show comparable outcomes in terms of complication rates and implant survival. However, in cases of severe alveolar ridge atrophy, extraoral donor sites (e.g., iliac crest or calvaria) may be required, which is associated with hospitalization, donor-site morbidity, and variable graft resorption rates. Therefore, alternative grafting solutions might be considered [7, 8]. Allogeneic bone blocks are generally considered as a favorable alternative to autogenous bone, even exhibiting a lower resorption rate (mean, 21.7%) than autogenous bone chips [9]. This approach can even be further refined by using CAD/CAM technologies to fabricate CABBs. By applying 3D planning based on preoperative cone-beam computed tomography (CBCT) scans, high-accuracy CABBs can be manufactured. This approach reduces the need for intraoperative manual adjustments, shortens surgical time, minimizes the risk of graft contamination, and reduces pressure on soft tissues due to an improved anatomical fit [10, 11]. In addition, the space between the grafts and recipient alveolar ridges is minimal, resulting in a larger contact surface, which is ideal for graft revascularization, cell migration and rapid integration [2, 12–14].

Although customized approaches are already used successfully in clinical practice, their accessibility remains relatively low due to high production costs, regulatory constraints, or personal preferences. To counteract these limitations, various authors have suggested preparing custom-made surgical devices in conjunction with conventional GBR approaches [15–17], manufacturing customized guides to shape a reinforced polytetrafluoroethylene (PTFE) membrane based on virtual bone augmentation plans. A 3D-printed augmented bone model and a replica of the virtually designed membrane were produced and used to pre-model and adapt the PTFE membrane extraorally. In a case report, our team presented a similar approach, using a membrane-cutting guide to shape the membrane according to digital augmentation plans. Although the two methods slightly differ, they both aim for more accurate treatment planning, shorter surgical duration, and a reduced complication rate.

Beyond accurate visualization of defect morphology and the manufacturing of customized surgical devices, 3D technology enables reverse planning, in which the desired prosthetic outcome drives surgical rehabilitation. In dental implantology, this concept has been well documented for several years [18]. Future dental implant positions are determined by the ideal implant-borne restoration, thus facilitating prosthetically driven implant placement. This method could be adapted to GBR procedures, in which the extent of the surgery is determined by the future ideal peri-implant hard tissue environment. While this approach has been briefly discussed in our previous case report, due to the limited sample size (*n* = 3), far-reaching conclusions could not be drawn.

While, in theory, any barrier membrane may be applied within the proposed method, properties such as form stability and resistance to fluid penetration make certain membranes more suitable. PTFE membranes, such as the previously utilized dense PTFE membrane (permamem^®^, botiss biomaterials GmbH, Zossen, Germany), have been demonstrated to be effective for the reconstruction of vertical alveolar ridge deficiencies. Indeed, sufficient volumetric hard tissue gain was even achieved in cases with membrane exposure [19].

Hence, this study aimed to present a prosthetically driven, reverse-planning method for the surgical rehabilitation of advanced vertical and horizonto-vertical alveolar ridge defects. Using a fully digital workflow, alveolar ridge augmentation was virtually simulated, and membrane-cutting guides were produced via additive manufacturing. The success of 3D-driven GBR was analyzed using volumetric subtraction.

## Materials and methods

### Study design

This retrospective study presents the linear and volumetric radiographic changes in 15 cases following vertical ridge augmentation using a 3D-driven reverse-planning method in conjunction with a split-thickness flap design and a tent-pole approach. Participants were treated at the Department of Periodontology, Semmelweis University, Budapest. The study protocol was approved by the Semmelweis University Regional and Institutional Committee of Science and Research Ethics (Approval Number: SE RKEB 145/2018). Surgical interventions were performed with the understanding and written informed consent of every participant. The study was conducted in full accordance with the ethical principles of the World Medical Association, Declaration of Helsinki for medical research involving human participants (2024 revision, published in 2025) [20]. Surgical procedures were performed by two expert surgeons (BM and PW), and CBCT segmentation and radiographic evaluations were performed by two independent examiners (DP and FBO). The current study followed the PROCESS guidelines [21], and the PROCESS checklist is included as a supplementary document to this manuscript (SD1).

### Patient selection

Patients with at least a single-tooth gap who required vertical augmentation to achieve optimal functional and aesthetic outcomes for implant-supported prosthetic rehabilitation were enrolled in the study. Alveolar ridge defects were classified according to the horizontal-vertical-combination (HVC) classification. Enrolled defects could either be classified as Class II (vertical: V) or Class III (combination: C) medium or large defects, respectively [22].

Exclusion criteria were as follows:

- Presence of relevant general medical conditions, such as previous irradiation therapy in the maxillo-facial area, uncontrolled diabetes, systemic steroid treatment, systemic bisphosphonate treatment, pregnant or lactating women.
- Smoking status: only non-smoking patients were enrolled.
- Periodontal status: untreated periodontitis with high levels of active pockets, full-mouth bleeding score (FMBS) ≥ 25% [23].
- Oral hygiene: full-mouth plaque score (FMPS)≥ 25% [24].

### Data acquisition and processing

#### CBCT imaging

CBCT images were taken prior to and 9 months following a vertical GBR procedure [25]. CBCT images were acquired with the following parameters:

- I-CAT FLX^®^ (KaVo Dental GmbH, Biberach an der Riß, Germany):
  - ◦ Voxel size = 300 μm.
  - ◦ Voltage = 120 kV.
  - ◦ Tube current = 36 mA.
  - ◦ Scan time = 8.9 s.
- Planmeca Viso^®^ (Planmeca, Helsinki, Finland):
  - ◦ Voxel size = 150 μm.
  - ◦ Voltage = 100 kV.
  - ◦ Tube current = 12 mA.
  - ◦ Scan time = 5.04–8 s.

#### Intraoral scanning and digital wax-up

Digital impressions of the lower and upper jaws and bite registration were taken utilizing an intraoral scanner (i700, Medit, MEDIT Corp., South Korea). Digital wax-ups were generated using these data to visualize the optimal positions of the future implant-borne prosthetic restoration.

#### Generating the virtual patient model

**Fig. 1:**
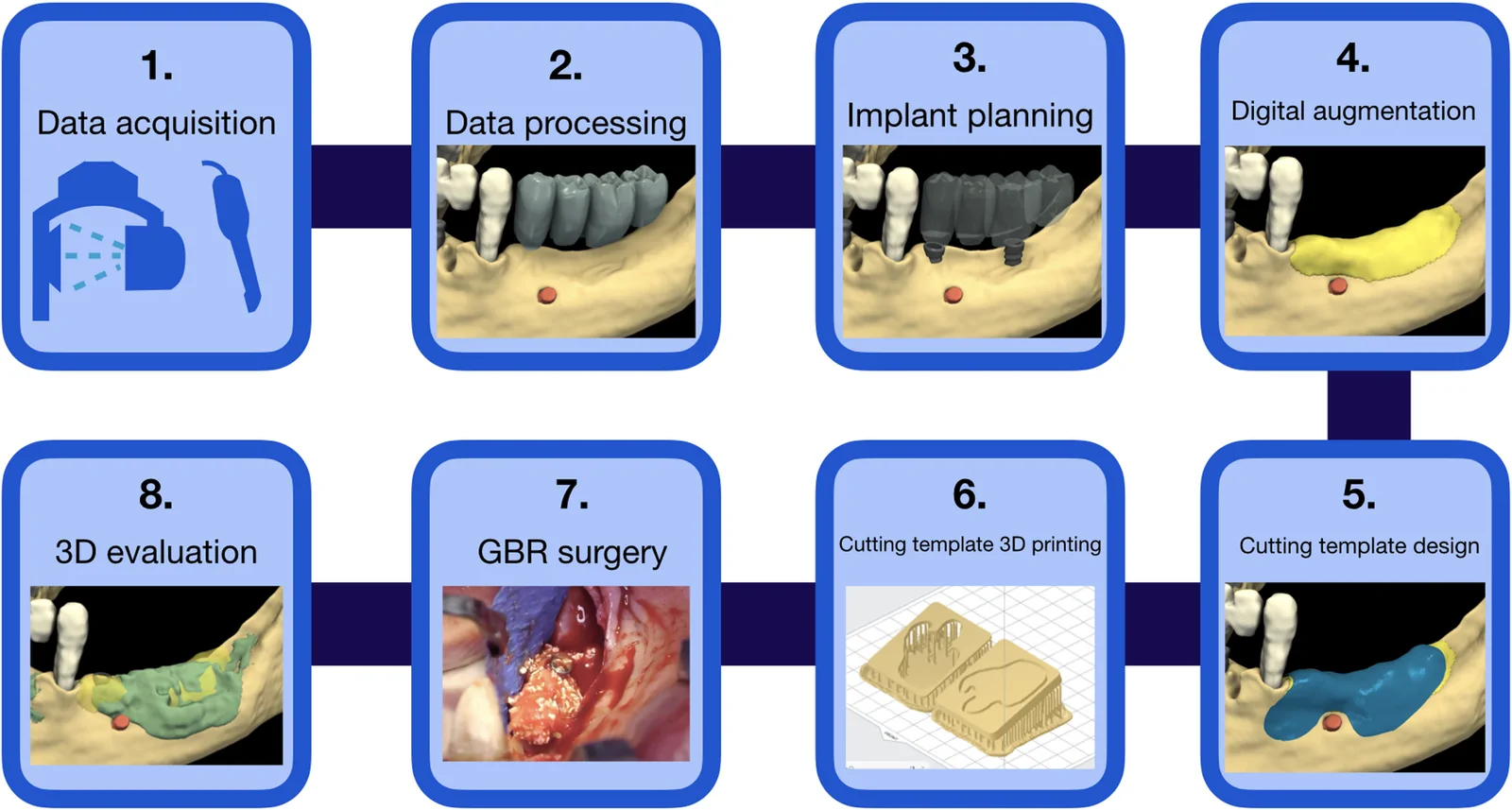
Digital reverse planning workflow

CBCT scans and digital wax-ups were imported into an open-source radiographic image processing software (3D Slicer, www.slicer.org) [26]. The segmentation was performed utilizing region-based semi-automatic method. The alveolar bone was segmented via region-growing [27–29], while the teeth were segmented using the watershed method [29]. Occasional errors were corrected with manual segmentation tools. As a result, virtual 3D models of relevant anatomical structures (i.e., teeth, alveolar bone and the inferior alveolar nerve) were acquired. A virtual patient model was generated by registering the segmented CBCT-derived model with the digital wax-up using a landmark-based registration method, thereby enabling the acquisition of a digital twin of the patient (Fig. 1A-B).

#### Augmentation planning

**Fig. 2:**
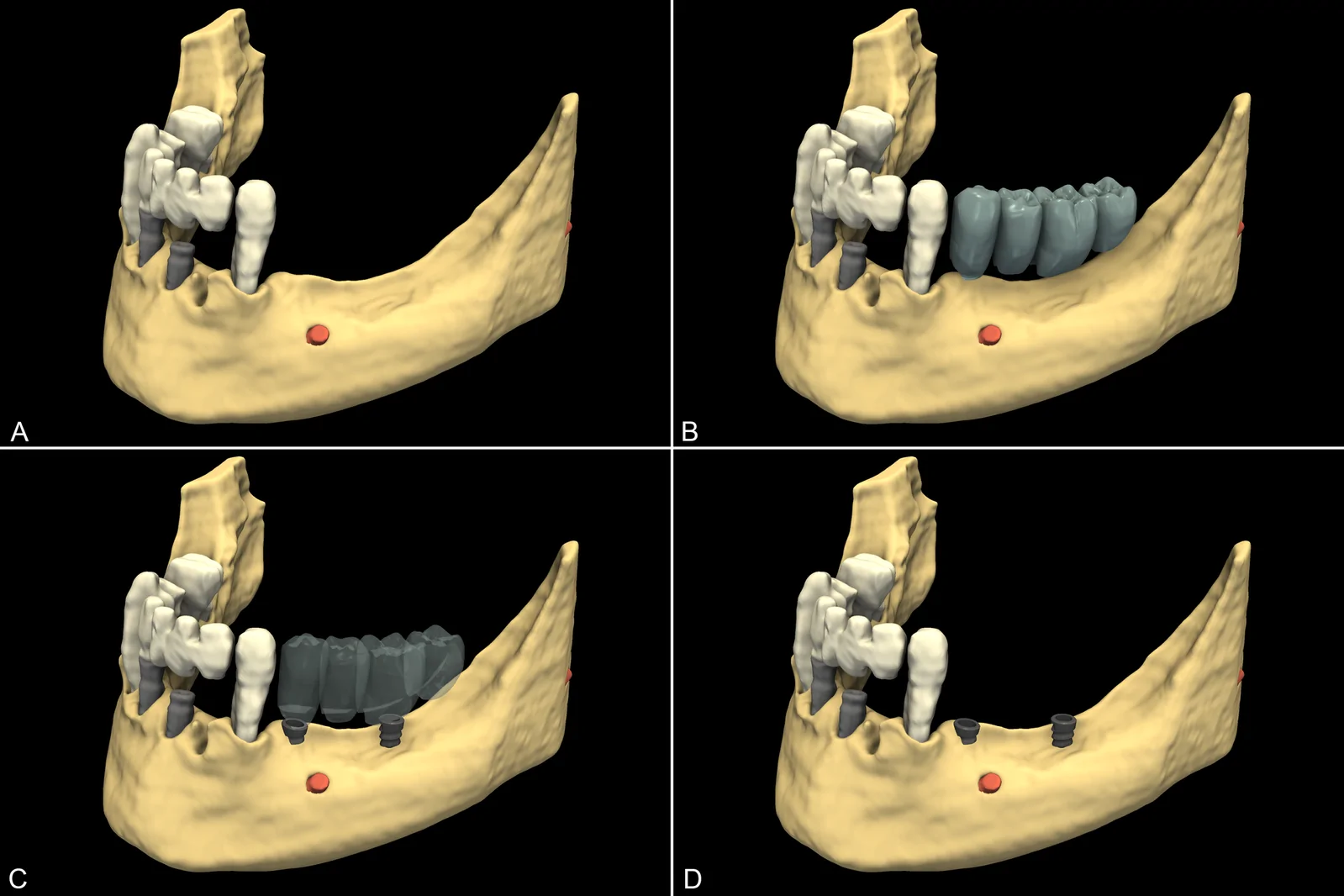
Digital prosthetically driven implant planning. **A** Baseline alveolar ridge configuration. **B** Digital wax-up. **C**–**D** Prosthetically driven implant positions

Using the virtual patient models, the hard- and soft-tissue environment of the edentulous ridge, in conjunction with the ideal prosthetic positions, was simulated (Fig. 1C-D). The extent of bone reconstruction was virtually planned based on the previously determined ideal implant positions. Virtual augmentation was performed by modifying the baseline alveolar ridge configuration with segmentation tools (i.e., sphere brush, smoothing; Fig. 2A). The digital augmentation volume was determined to ensure that the planned implants were fully supported by adequate bone in all directions. Implant diameters were standardized at 4 mm, while implant length was set at 8–10 mm.

**Fig. 3:**
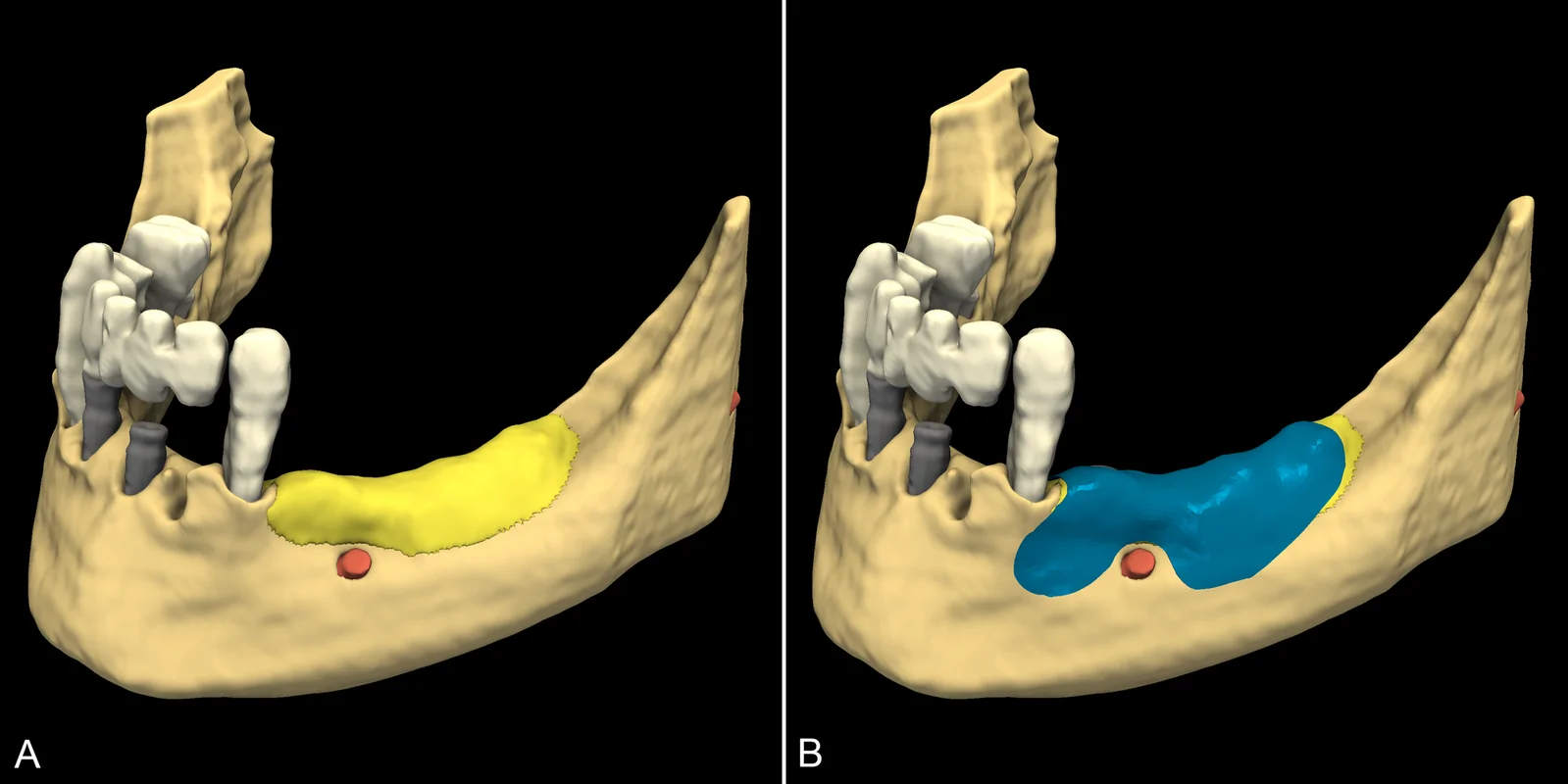
Digital augmentation planning. **A** Desired hard tissue gain (in yellow) based on previously determined implant positions. **B** Digitally designed shape of the utilized barrier membrane (permamem®, Botiss Biomaterials GmbH, Zossen, Germany)

The required size and 3D shape of the barrier membrane were defined based on the digitally reconstructed ideal contour of the alveolar ridge. The shape of the membrane was traced on the reconstructed alveolar bone using the *closed curve mark-up* tool. Subsequently, the closed surface model of the barrier membrane was generated using the *Baffle planner* module of the SlicerHeart extension [30] (Fig. 2B). The membrane geometry was then digitally flattened, and a two-dimensional cutting template was parametrically modeled in Fusion 360 (Autodesk, San Francisco, CA, USA; Fig. 3A). The template was fabricated using additive manufacturing with a stereolithography (SLA) 3D printer (Form 3B^®^, Formlabs, Somerville, MA, USA) and a biocompatible surgical-grade resin (Dental SG V2^®^, Formlabs, Somerville, MA, USA) (Fig. 3B).

### Surgical procedure

**Fig. 4:**
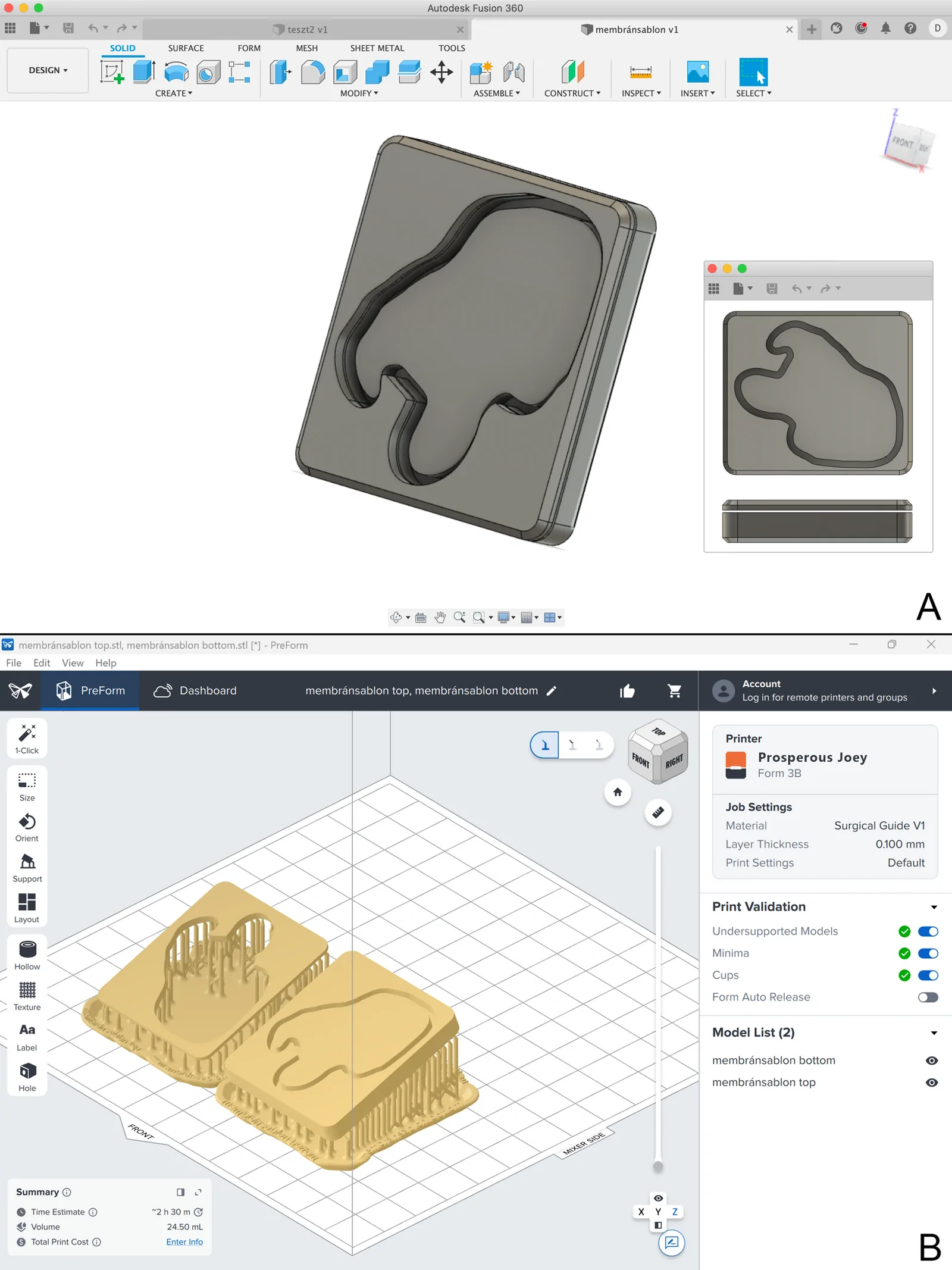
Computer-aided design and manufacturing (CAD/CAM) of the membrane-cutting templates. **A** Parametric CAD modeling of the membrane-cutting guide (Fusion 360®, Autodesk). **B** Cutting guide prepared for stereolithographic (SLA) 3D printing (Preform®, Formlabs)Figure 5: Shaping of the barrier membrane

A non-resorbable dense PTFE membrane (permamem^®^, botiss biomaterials GmbH, Zossen, Germany) was shaped using the previously designed, manufactured and sterilized membrane-cutting guide (Fig. 4).

**Fig. 5:**
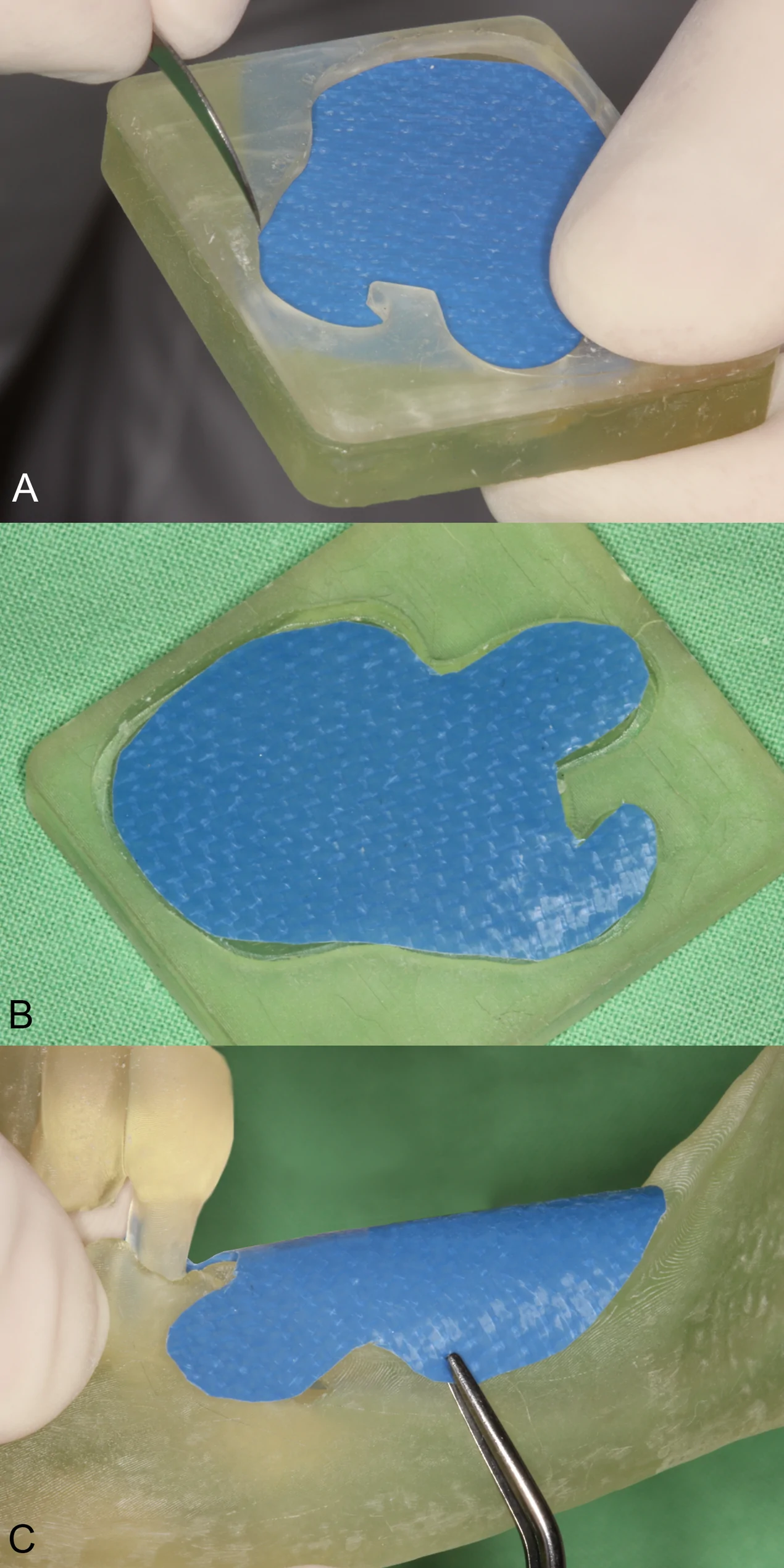
Shaping of the barrier membrane. **A**–**B** Cutting the membrane with a no. 12 blade using the 3D-printed, sterilized cutting template. **C** Testing the fit on a 3D-printed, sterilized anatomical model

Vertical GBR was performed using a split-thickness flap design as previously described by Windisch et al. [19, 31, 32]. Under local anesthesia (Ultracain DS Forte, Sanofi-Aventis), a horizontally extended midcrestal incision was made along the edentulous ridge to facilitate flap mobilization without the use of vertical incisions. On the lingual/palatal aspect, a full-thickness flap was elevated to allow buccal displacement and tension-free wound closure. On the buccal aspect, a two-layer flap design was utilized by first dissecting the mucosal layer from the periosteum and then bluntly elevating the periosteal layer from the underlying bone (Fig. 5A–B).

Following flap elevation, autogenous bone chips were harvested from the same surgical incision using a disposable bone collector (SafeScraper Twist^®^, Meta, Reggio Emilia, Italy). The harvested bone was mixed with a bovine-derived xenograft (cerabone^®^, botiss biomaterials GmbH, Zossen, Germany) in a 1:1 ratio to obtain a composite graft [33] (Fig. 5C). Tenting screws (Pro-Fix™ Precision Fixation System, Osteogenics, Lubbock, TX, USA) were placed to support the membrane and ensure adequate space maintenance (Fig. 5D).

The extraorally shaped membrane was secured with titanium fixation screws (Pro-Fix Precision Membrane Fixation System^®^, Osteogenics Biomedical, Lubbock, TX, USA) or pins (Titan pinset^®^, botiss biomaterials GmbH, Zossen, Germany) on the lingual aspect. The composite graft was packed around the tenting screws, after which the membrane was secured on the buccal aspect (Fig. 5E–F). Primary wound closure was achieved using a double-layer suturing technique (Fig. 5G–H).

### Radiographic evaluation

Follow-up CBCT scans were obtained 9 months after surgery. These scans were segmented similarly to the baseline CBCT scans and aligned to the baseline CBCT images using a voxel-intensity-based registration method [34]. Following superimposition, volumetric and morphological changes in hard tissue were assessed by comparing baseline and follow-up 3D models.

#### Linear radiographic measurements

**Fig. 6:**
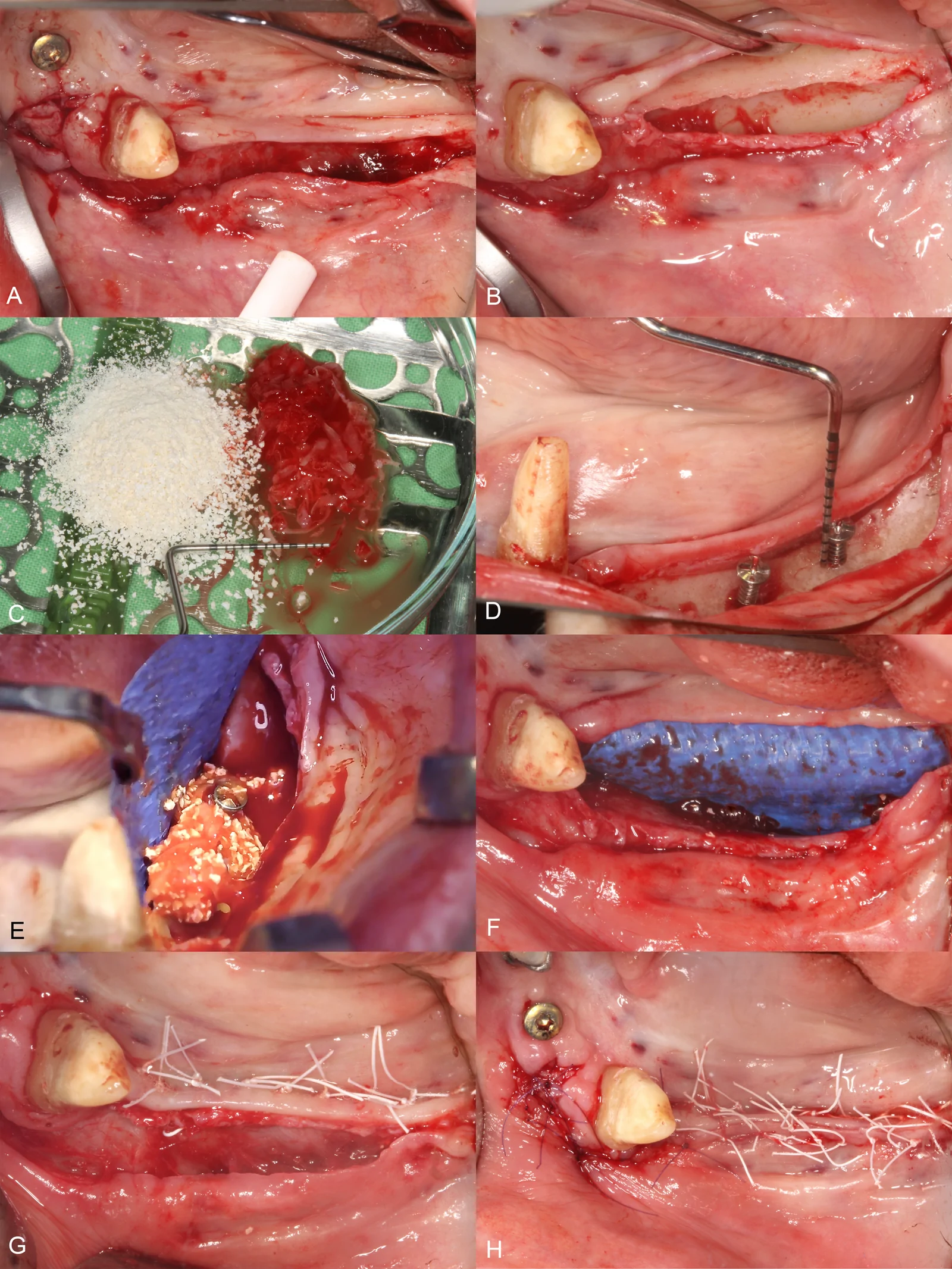
Guided bone regeneration procedure [19]. **A** Split-thickness flap preparation: Elevating the mucosal layer. **B** Split-thickness flap preparation: Blunt dissection of the periosteal layer. **C** Composite graft with a 1:1 ratio of bovine-derived xenograft (cerabone®, Botiss Biomaterials GmbH, Zossen, Germany) and autogenous bone chips. **D** Placement of the tenting screws. **E** Lingual membrane fixation and packing of the graft material. **F** Buccal membrane fixation. **G** Double-layer wound closure: periosteal layer. **H** Double-layer wound closure: mucosal layer

**Fig. 7:**
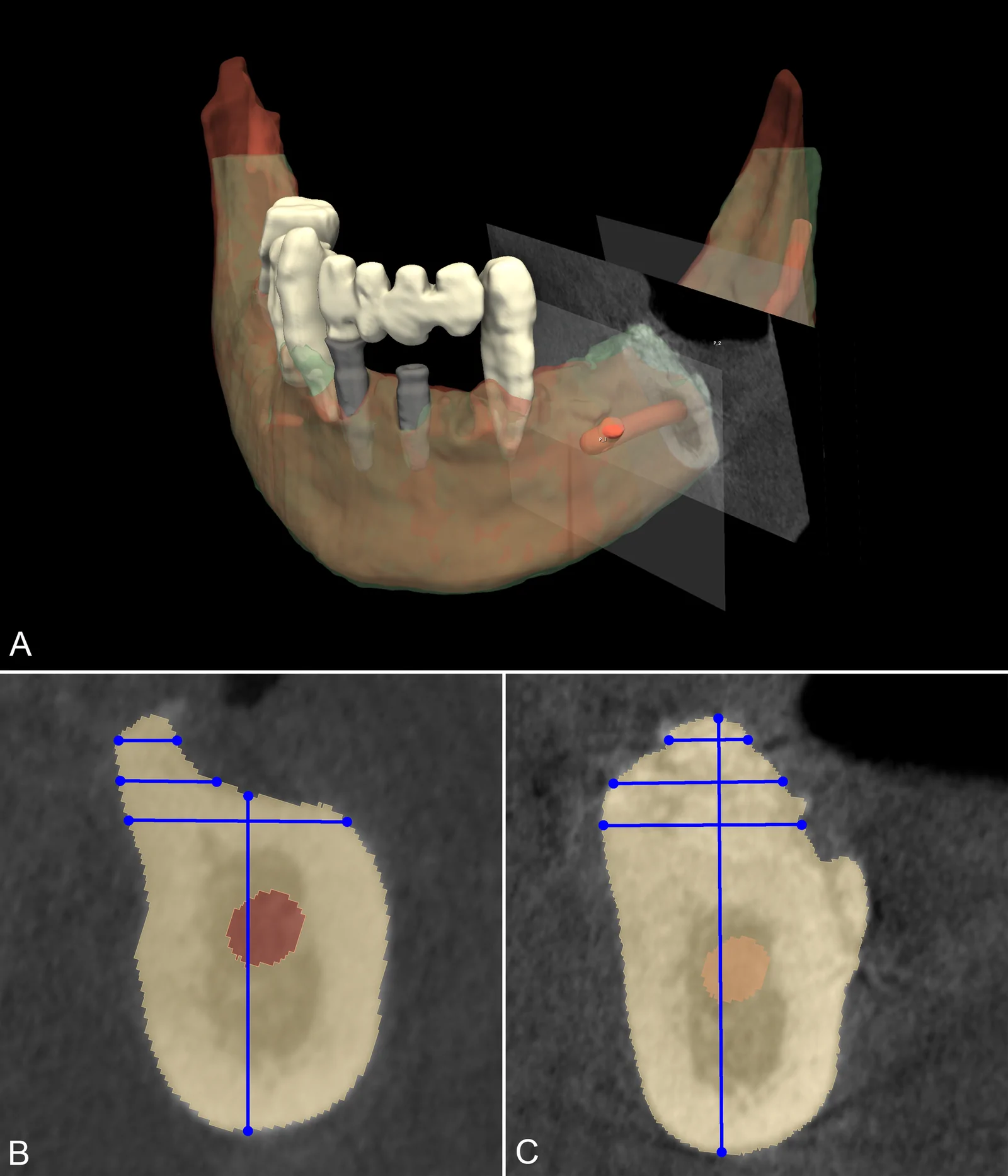
Linear measurements of the alveolar ridge at baseline and 9-month follow-up. **A** Vertical and horizontal measurements recorded at the mesial, central, and distal aspects of the surgical site (central measurement site shown). **B** Baseline vertical and horizontal measurements. **C** 9-month follow-up vertical- and horizontal measurements

The primary outcome of the study was to evaluate vertical linear changes from baseline to 9-month follow-up. For all linear measurements, CBCT images were reoriented so that the sagittal axis became parallel, and the coronal axis became perpendicular to the alveolar ridge at the surgical area. Vertical measurements were made parallel to the coronal axis and were recorded at three locations within the surgical site (mesial, central, and distal) between the midcrestal line and the base of the mandible or the maxillary alveolar process. Horizontal measurements were exactly perpendicular to the vertical measurements, matching the sagittal axis of the reoriented CBCT dataset. Horizontal dimensional changes were evaluated at the same measurement sites at 1, 3, and 5 mm apical to the alveolar crest (Fig. 6). Vertical and linear measurements taken at multiple measurement sites were used to determine the mean vertical and horizontal bone dimensions at each specific site. Thereby, it was assessed whether a sufficient amount of hard tissue was obtained to allow placement of an implant with the previously determined dimensions (Fig. 7).

#### Volumetric analysis

**Fig. 8:**
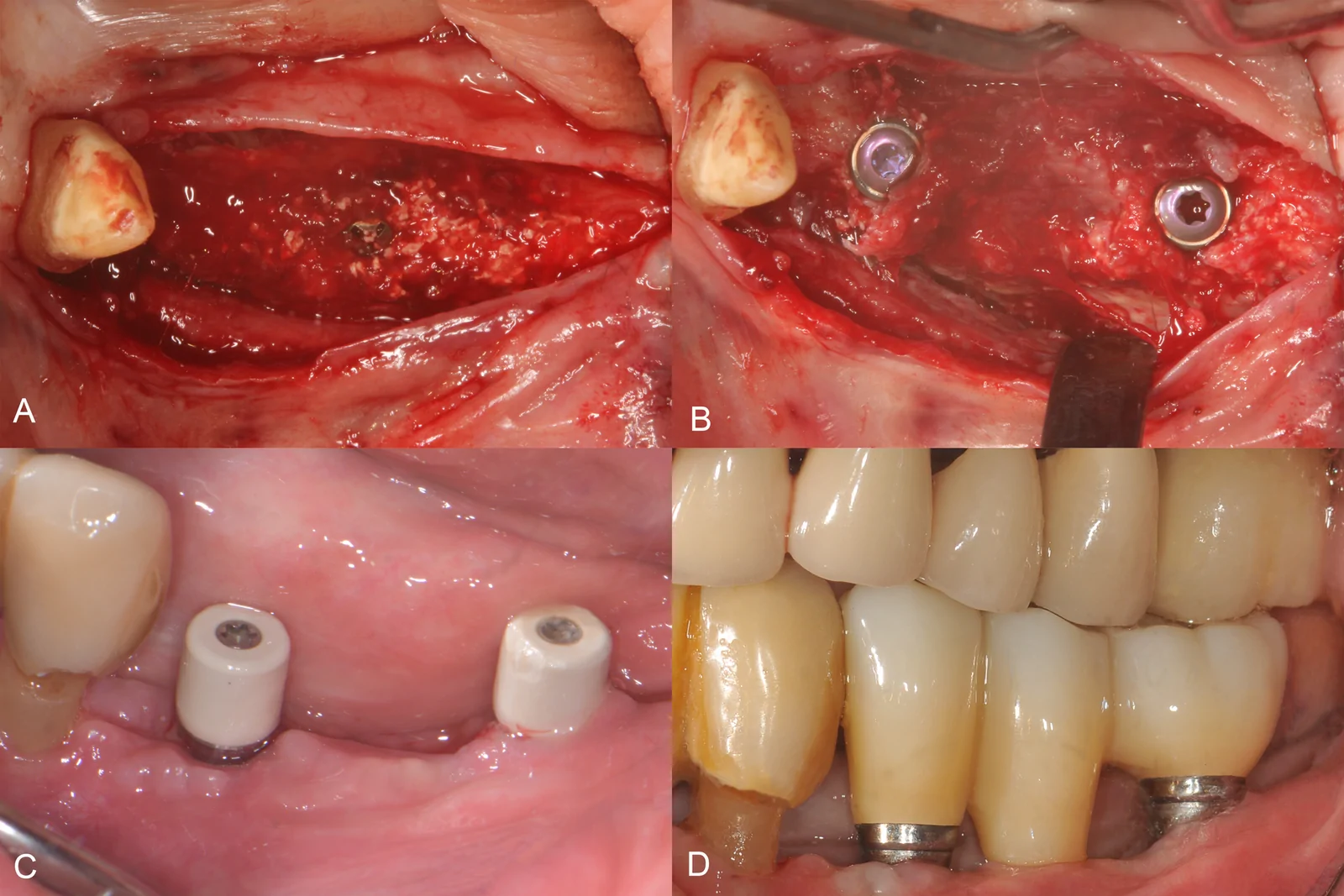
Alveolar ridge configuration at re-entry. **A** Alveolar ridge at re-entry (9-month follow-up after augmentation). **B** Implant positions. **C** Peri-implant soft tissue condition 2 weeks after abutment connection. **D** Final prosthetic solution

**Fig. 9:**
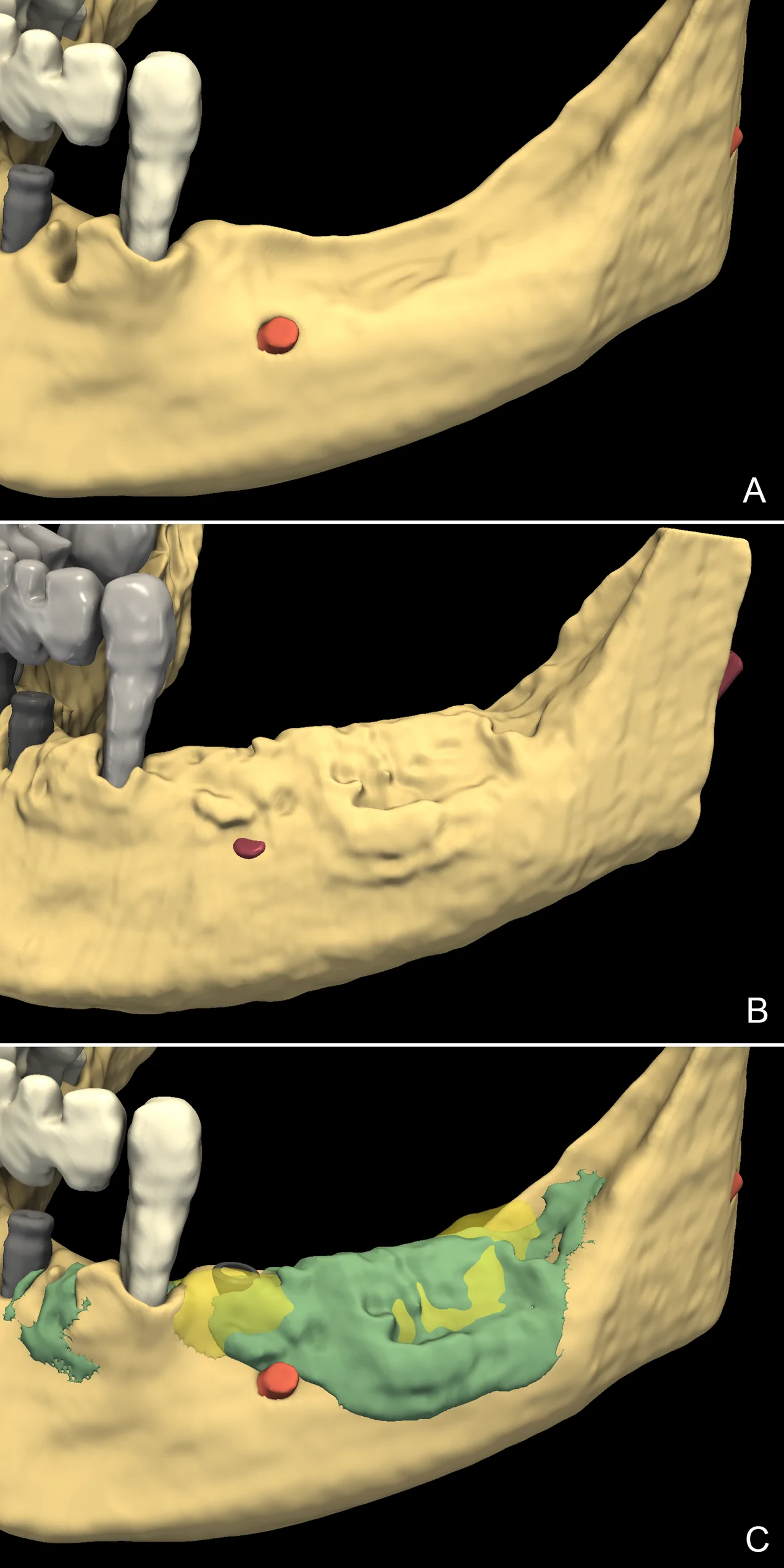
Volumetric hard tissue evaluation. **A** Baseline alveolar ridge morphology. **B** 9-month follow-up alveolar ridge morphology. **C** Preoperatively planned hard tissue volume shown in yellow, 9-month volumetric hard tissue gain shown in green

In addition to linear assessment, volumetric evaluation was also performed by subtracting the baseline segmentations from the 9-month follow-up segmentations. The difference between the two models reflected the hard tissue changes achieved with vertical GBR. Volumetric differences were calculated in the Segment statistics module of 3D Slicer and expressed in mm^3^. In addition to actual hard tissue changes, the planned hard tissue volume was also determined. Similarity between the digitally planned volume and the actual hard tissue gain was assessed to indirectly evaluate the impact of the digital planning process (Fig. 8 and Fig. 9).

#### Validation of augmentation efficacy: volume-to-length ratio

**Fig. 10:**
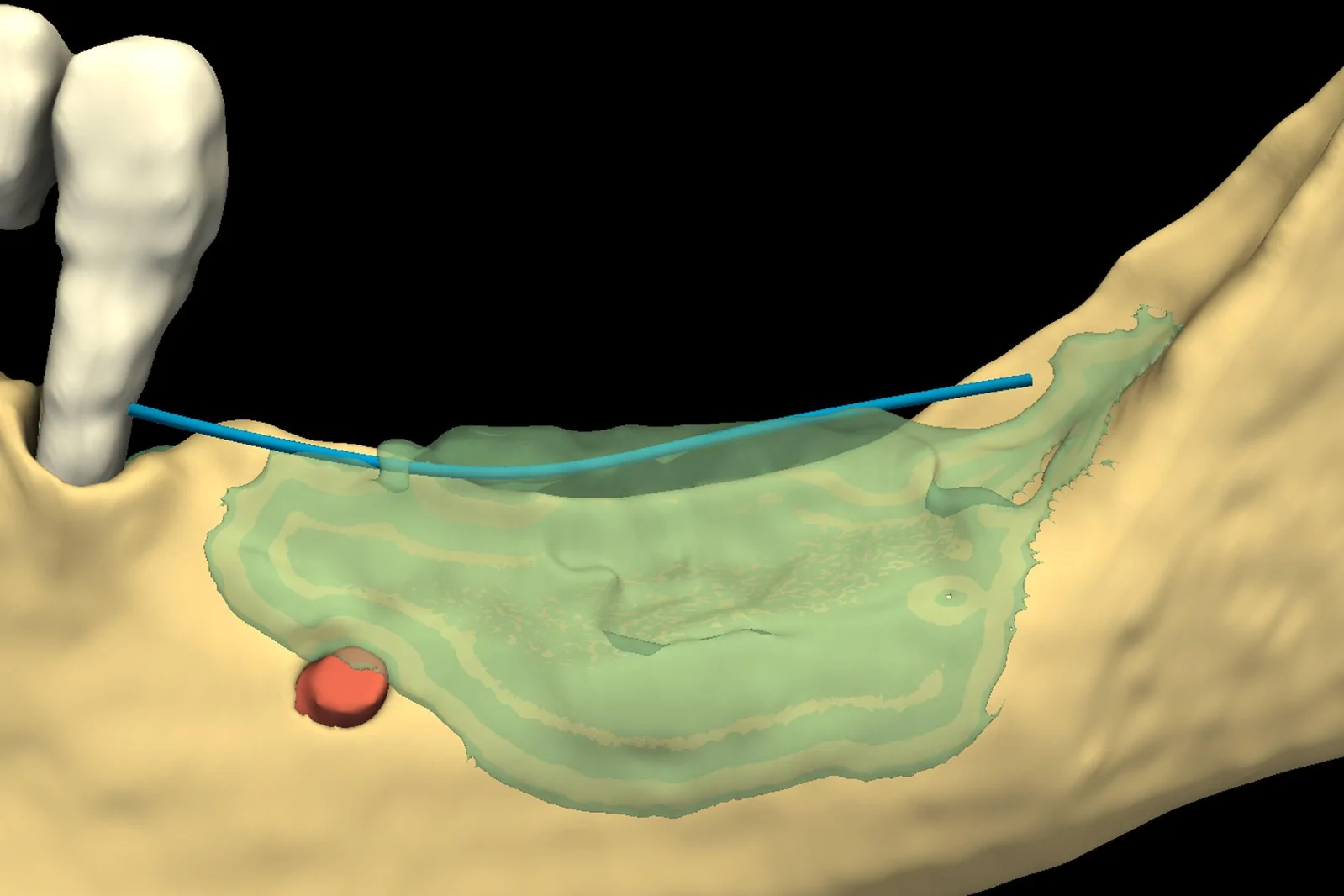
Augmentation efficacy. Blue open-curve mark-up measures the mesio-distal extension of the augmentation site; volumetric hard tissue gain is visualized in green. Augmentation efficacy is calculated by dividing the volumetric hard tissue gain (mm^3^) by the length of the augmentation area (mm)

To assess augmentation efficacy independent of surgical area size, a volume-to-length ratio was defined as a standardized outcome measure, representing the average volumetric hard tissue gain per unit length of the augmented area. The linear extent of augmentation was determined using an open-curve mark-up, spanning the surgical site from its most mesial to most distal limits (Fig. 10). The volume-to-length ratio was calculated as:

$$\:\frac{volumetric\:hard\:tissue\:gain\:\left(mm3\right)}{length\:of\:augmented\:area\:\left(mm\right)}$$

### Calibration

To assess segmentation consistency, intra- and inter-rater reliability were evaluated within a single rater and between two independent raters. Segmentation consistency was evaluated using standard segmentation performance metrics: dice similarity coefficient (DSC), intersection-over-union (IoU), and the 95th percentile of the Hausdorff distance (HD95). For linear measurements, intra-rater reliability was evaluated during two calibration sessions, during which a single rater performed linear measurements on segmentations independent of the study sample. The intraclass correlation coefficient (ICC) was calculated to assess intra-rater reliability for linear measurements.

### Statistical analysis

Descriptive statistics were used to summarize the data, and the results were presented as mean, standard deviation, median, minimum, and maximum. The normality of the variable’s distribution was assessed using the Shapiro–Wilk test. Parametric tests were applied to normally distributed variables, while non-parametric tests were used when normality assumptions were violated. Paired comparisons were analyzed using the paired Student’s t-test or, when normality was not met, the Wilcoxon signed-rank test. A p-value of less than 0.05 was considered statistically significant. Correlation between planned volume and 9-month volumetric changes was assessed using Spearman’s rho (*ρ*). All statistical calculations were performed using the STATA software package (release 18; Stata Corp LLC, College Station, TX, USA).

## Results

### Patient demographics

Fifteen patients participated in the current investigation. In eight cases, alveolar ridge defects were classified as vertical deficiencies, while in seven cases, combination defects were identified. Four of the surgical sites were in the upper front region, and 11 were in the lower lateral region. Due to inadequate baseline hard tissue dimensions, simultaneous augmentation and implant placement were not feasible in any of the cases. Twelve of the 15 participants were female, and three were male. The mean age was 59.33 ± 14.82 years. Table 1 summarizes demographic and surgical site-related data.

**Table 1:** Patient demographics and site characteristics

Case	Sex (^1^F/^2^M)	Age	Localization	Surgical area by tooth
1	F	66	Post. Mand.	44, 45, 46, 47
2	F	65	Post. Mand.	44, 45, 46
3	F	65	Post. Mand.	34, 35, 36
4	M	59	Post. Mand.	34, 35, 36, 37
5	F	78	Post. Mand.	44, 45, 46
6	M	58	Post. Mand.	44, 45, 47
7	F	78	Post. Mand.	36, 37
8	F	76	Post. Mand.	34, 35, 36
9	F	56	Post. Mand.	35, 36, 37
10	F	55	Post. Mand.	44, 45, 46, 47
11	F	40	Ant. Max.	21, 11, 12, 13
12	M	45	Ant. Max.	11, 21
13	F	26	Ant. Max.	11, 12
14	F	74	Ant. Max.	21, 22
15	F	52	Post. Mand.	35, 36, 37

^1^Female

^2^Male

### Consistency

The mean DSC, IoU, and HD95 values between the segmentations of rater 1 were 0.97 ± 0.01, 0.95 ± 0.01, and 0.27 mm ± 0.08 mm, respectively. The same metrics for segmentation by rater 2 averaged 0.96 ± 0.02 (DSC), 0.94 ± 0.01 (IoU), and 0.28 mm ± 0.11 mm (HD95). Mean DSC, IoU, and HD95 values determining the inter-rater reliability were 0.92 ± 0.02, 0.90 ± 0.03, and 0.31 mm ± 0.13 mm. High consistency was observed for both intra- and inter-rater segmentations, indicating strong agreement within and between raters. A similarly strong positive correlation was observed between two sets of linear measurements obtained by a single rater, with an ICC of 0.96 (*p* < 0.0001).

### Changes in linear alveolar ridge dimensions

Baseline and 9-month vertical hard tissue dimensions at the most mesial point of the surgical site averaged 17.04 mm ± 5.17 mm and 21.32 mm ± 4.13 mm, respectively. At the center of the augmented site, they changed from 15.70 mm ± 4.34 mm at baseline to 19.96 mm ± 3.83 mm at 9-month follow-up. Regarding the most distal point of the site, the mean vertical hard tissue dimensions were 15.25 mm ± 3.52 mm (baseline) and 18.65 mm ± 3.57 mm (9-month follow-up). These vertical dimensional changes were found to be statistically significant (mesial: *p* = 0.007; central: *p* = 0.007; distal: *p* = 0.004).

Baseline horizontal alveolar ridge dimensions measured at 1, 3, and 5 mm apical to the alveolar crest at the most mesial point of the surgical area averaged 6.17 mm ± 2.59 mm, 7.40 mm ± 2.26 mm and 8.20 mm ± 2.15 mm, whereas 9-month follow-up values at the same measurement levels were 5.18 mm ± 1.16 mm, 7.65 mm ± 1.17 mm and 9.06 mm ± 1.27 mm, respectively. This difference was found to be statistically significant (*p* = 0.016) at 1 mm, non-significant (*p* = 0.627) at 3 mm and borderline non-significant (*p* = 0.054) at 5 mm apical from the alveolar crest. At the center of the surgical site, the mean horizontal changes at the three measurement levels were 6.50 mm ± 2.12 mm, 8.58 mm ± 2.52 mm, and 9.23 mm ± 2.56 mm, while values following GBR were 5.33 mm ± 1.41 mm, 7.67 mm ± 1.52 mm and 8.90 mm ± 1.81 mm. This difference was found to be statistically significant (*p* = 0.034) at 1 mm, borderline non-significant (*p* = 0.058) at 3 mm, and non-significant (*p* = 0.644) at 5 mm apical from the alveolar crest. Baseline horizontal changes at the most distal point of the surgical site at 1, 3, and 5 mm apical to the crest were 8.38 mm ± 3.21 mm, 9.79 mm ± 3.1 mm, and 10.03 mm ± 2.89 mm, respectively. In contrast, the follow-up mean values were 5.82 mm ± 1.60 mm, 8.71 mm ± 1.91 mm, and 9.99 mm ± 2.08 mm, respectively. This difference was found to be statistically significant (*p* = 0.007) at 1 mm, borderline significant (*p* = 0.043) at 3 mm, and non-significant (*p* = 0.926) at 5 mm apical from the alveolar crest. Tables 2 and 3 summarize the linear measurement values.

**Table 2:** Baseline and follow-up vertical alveolar ridge dimensions

Case	Vertical dimensions (mm)					
	**Baseline**			**Follow-up**		
	**Mesial**	**Middle**	**Distal**	**Mesial**	**Middle**	**Distal**
1	9.83	14.95	15.96	21.33	18.06	17.78
2	22.92	16.67	13.46	24.39	20.37	18.05
3	19.98	15.83	14.49	23.11	20.53	17.44
4	17.86	20.67	21.95	25.24	24.40	18.62
5	22.96	18.02	14.95	23.00	20.96	14.98
6	18.08	17.79	19.12	21.50	21.42	22.83
7	18.77	17.01	15.64	21.20	22.39	21.73
8	17.39	15.74	14.89	21.95	21.48	19.90
9	21.93	20.79	19.48	23.48	22.40	22.63
10	17.38	17.36	14.53	25.45	24.68	23.56
11	17.95	15.87	14.57	23.54	20.48	19.27
12	9.37	9.11	8.36	12.44	12.83	13.51
13	16.62	13.47	12.17	18.39	16.39	13.65
14	4.89	3.79	10.55	11.78	11.31	13.34
15	19.81	18.36	18.61	22.93	21.64	22.41
Mean ± St. Dev.	17.05 ± 5.17	15.70 ± 4.34	15.28 ± 3.52	21.32 ± 4.13	19.96 ± 3.83	18.65 ± 3.57

**Table 3:** Baseline and follow-up horizontal linear alveolar ridge dimensions

Case	Horizontal dimensions measured at different levels apical to the alveolar crest (mm)																			
	Mesial						Mid								Distal					
	1 mm		3 mm		5 mm		1 mm			3 mm			5 mm		1 mm		3 mm		5 mm	
	Pre^1^	Post^2^	Pre^1^	Post^2^	Pre^1^	Post^2^		Pre^1^	Post^2^		Pre^1^	Post^2^	Pre^1^	Post^2^	Pre^1^	Post^2^	Pre^1^	Post^2^	Pre^1^	Post^2^
1	9.50	3.22	8.91	7.38	8.45	9.15		5.96	5.81		8.20	8.08	8.44	8.56	5.53	5.79	7.06	8.43	8.00	8.54
2	2.22	5.26	4.54	7.39	6.57	8.79		5.60	5.47		8.95	8.27	10.46	10.11	12.39	7.05	11.51	10.56	10.28	12.43
3	4.03	6.61	8.02	8.06	8.34	10.27		7.83	7.76		10.82	8.71	10.04	10.36	10.59	9.96	10.04	10.14	9.48	9.93
4	10.8	6.41	11.46	8.65	11.70	10.02		7.67	6.22		10.20	8.42	12.55	10.40	9.30	5.68	12.71	8.39	13.38	11.80
5	2.48	4.66	3.72	7.58	5.71	8.08		5.02	5.10		9.12	7.01	10.28	7.77	11.02	5.03	13.92	11.26	13.50	12.81
6	8.27	7.22	10.42	9.69	11.72	11.02		9.31	7.64		12.84	10.12	14.13	11.69	10.18	6.66	13.75	9.99	14.89	12.37
7	7.60	4.85	8.45	9.48	9.57	10.49		7.60	4.25		10.14	8.18	10.18	9.29	12.57	6.91	12.39	8.91	12.32	9.73
8	6.85	5.41	7.80	6.93	8.72	7.77		8.34	4.60		9.52	7.56	9.40	8.97	10.00	5.71	10.34	8.48	10.88	10.11
9	5.27	5.17	6.70	7.29	7.27	7.29		6.00	6.10		9.03	8.52	9.47	9.67	8.62	5.34	11.00	10.01	9.73	11.34
10	6.83	6.33	8.12	8.34	7.79	10.31		6.96	6.00		7.21	8.29	6.87	9.87	9.11	6.28	9.55	10.43	8.81	10.14
11	4.26	4.71	5.49	7.54	6.35	8.68		3.63	3.42		5.13	6.34	6.66	7.73	4.43	3.80	7.80	6.91	9.01	8.45
12	5.16	4.24	7.18	6.13	10.03	8.29		3.43	5.25		6.03	7.35	7.41	6.63	3.92	4.42	6.40	6.76	9.14	8.61
13	3.77	4.55	3.66	6.29	3.87	6.71		2.61	3.31		2.72	4.75	3.41	6.54	3.11	3.25	3.67	5.58	3.52	6.36
14	9.22	3.26	7.91	5.52	7.42	9.27		8.06	3.13		8.11	4.48	8.47	5.75	4.54	4.90	5.43	4.97	6.51	6.10
15	6.38	5.79	8.59	8.44	9.55	9.81		9.44	5.91		10.60	9.06	10.53	11.62	10.60	6.59	11.34	9.94	11.06	11.19
Mean ± St. dev.	6.17 ± 2.59	5.18 ± 1.16	7.40 ± 2.26	7.65 ± 1.17	8.20 ± 2.15	9.06 ± 1.27		6.5 ± 2.12	5.33 ± 1.41		8.58 ± 2.52	7.67± 1.52	9.23 ±2. 56	8.90 ± 1.81	8.38 ± 3.21	5.82± 1.60	9.79 ±3.1	8. 71 ±1.91	10.03 ± 2.89	9.99±2.08

Pre^1^ Preoperative measurements

Post^2^ Postoperative (9-month follow-up) measurements

### Volumetric changes and augmentation efficacy

Following segmentation and superimposition of the baseline and 9-month follow-up CBCTs, volumetric hard tissue alterations were assessed. The mean hard tissue gain following vertical ridge augmentation was 755.33 mm^3^ ± 411.22 mm^3^. Based on the prosthetically ideal implant position, the planned augmentation volume averaged at 757.50 mm^3^ ± 417.78 mm^3^. Using the Wilcoxon matched-pairs signed-rank test, no statistically significant difference (*p* = 0.649) in volume was observed compared with the 9-month follow-up results. Additionally, a strong, statistically significant positive correlation was identified between the planned and 9-month outcome volumes (Spearman’s ρ = 0.825, *p* = 0.0004).

The mean extent of the augmented sites was 26.09 mm ± 7.13 mm. Subsequently, the volume-to-length ratio, which was used to assess the efficacy of GBR, averaged 20.13 mm^3^/mm ± 15.21 mm^3^/mm, as shown in Table 4.

**Table 4:** Volumetric hard tissue alterations and efficacy of the vertical ridge augmentation

Case	Planned volume (mm^3^)	Volumetric gain (mm^3^)	Mesio-distal extent (mm)	Efficacy
1	851.23	987.96	30.00	32.93
2	652.21	946.00	30.00	31.53
3	550.76	810.00	30.00	27.00
4	563.67	618.27	28.80	21.47
5	309.87	339.28	34.94	9.71
6	1048.63	928.06	33.00	28.12
7	523.56	553.64	25.00	22.15
8	888.45	808.91	32.20	25.12
9	552.44	351.56	28.27	12.44
10	1844.12	1835.00	26.50	69.25
11	1487.78	1317.19	26.24	50.20
12	523.23	509.82	16.24	31.39
13	523.67	446.92	9.70	46.07
14	453.23	374.17	15.00	24.94
15	589.67	503.18	25.49	19.74
Mean ± St. dev.	757.50 ± 417.78	755.33 ± 411.22	26.09 ± 7.13	20.13 ± 15.21

### Complications and implant-related outcomes

Moderate postoperative swelling was observed following ridge augmentation procedures. Membrane exposure occurred in four of the 15 cases (cases #1, #5, #11, and #12); however, no superinfection of the augmented sites or substantial differences outcomes could be detected. In one case, the exposed area was trimmed, and secondary epithelialization occurred after 1 week. In three cases, secondary epithelialization did not occur and membranes had to be removed between the 6th and 12th postoperative week. Despite membrane exposures, horizontal and vertical dimension of hard tissue was adequate to accommodate implants of standard diameter and length in all cases. No secondary augmentation procedures were required.

In all 15 augmented sites, implants were placed in the previously planned prosthetically ideal positions. None of the cases required additional bone augmentation at the time of implant placement. Insertion torque values ranged between 25 and 35 Ncm. All implants healed uneventfully and were subsequently restored prosthetically. Table 5 shows the implant-related outcome.

**Table 5:** Implant related outcomes

Case	Implant placement feasibility	Number of the placed implants	Insertion torque (ncm)	Implant sizes
1	Yes	2	35, 30	4.1 × 10, 4.1 × 10
2	Yes	2	35, 35	4.1 × 10, 4.1 × 10
3	Yes	2	35, 35	4.1 × 10, 4.1 × 10
4	Yes	2	35, 25	4.1 × 10, 4.1 × 8
5	Yes	2	35, 35	4.1 × 10, 4.1 × 10
6	Yes	3	35, 30, 35	4.1 × 10, 4.1 × 8, 4.1 × 8
7	Yes	2	35, 25	4.1 × 10, 4.1 × 8
8	Yes	2	25, 35	4.1 × 10, 4.1 × 10
9	Yes	2	35, 25	4.1 × 10, 4.1 × 8
10	Yes	3	30, 35, 35	4.1 × 10, 4.1 × 10
11	Yes	2	35, 35	4.1 × 10, 3.3 × 10, 4,1 × 12
12	Yes	2	35, 30	4.1 × 10, 3.3 × 10
13	Yes	2	25, 25	4.1 × 10, 3.3 × 10
14	Yes	2	25, 25	4.1 × 10, 3.3 × 10
15	Yes	2	25, 30	4.1 × 10, 4,1 × 10

## Discussion

This retrospective feasibility study evaluated a fully digital, reverse-planning workflow for the reconstruction of vertical and combination alveolar ridge defects [22] using dense PTFE membranes shaped with digitally fabricated cutting templates. By integrating CBCT-based segmentation, prosthetically driven planning, and additive manufacturing, the proposed approach aimed to enhance the predictability of GBR procedures. Augmentation outcomes were assessed using linear and volumetric radiographic analyses, including a volume-to-length ratio to normalize hard tissue gain relative to the extent of the surgical site.

The concept of digitally assisted membrane shaping was previously described by Cucchi et al. [15, 16], who shaped reinforced PTFE membranes based on virtual augmentation plans. Their approach similarly relied on extraoral pre-shaping of the membrane using 3D-printed reference models to reduce intraoperative manipulation and improve graft stability. While the methodological principles are comparable, the present workflow differs by emphasizing reverse prosthetic planning and a digitally flattened membrane-cutting guide rather than shaping the membrane directly on an anatomical model. In their article, the mean vertical linear bone gain at the center of the augmented sites was 5.50 mm. In the present study, the corresponding vertical gain averaged 4.26 mm, which was lower than that reported by Cucchi et al. but remained within a clinically relevant range, given differences in study design, defect morphology, and augmentation strategy. In a previous case study, Soares et al. [35] reported an overall augmentation efficacy of 0.037 ± 0.011 cm³/mm (≈ 37 ± 11 mm³/mm) using CTMs, which is higher than the value observed in the present study, potentially reflecting differences in baseline defect severity. However, the volume-to-length ratio is not a standard metric for assessing the success of alveolar ridge augmentation. This parameter was included to provide a more standardized assessment of volumetric gain by accounting for the mesiodistal extent of the augmented region. Since sites involving multiple teeth inherently yield greater absolute volume gain, normalization to the augmentation length allows for a more meaningful comparison between cases.

When compared with CTMs or CABBs [2, 3], the current digital approach may facilitate broader clinical acceptance, particularly among clinicians less experienced with rigid barriers or block grafts. Furthermore, the current approach does not require dedicated industrial manufacturing processes, specialized regulatory pathways, or material-specific workflows, thereby improving accessibility. An inherent limitation of the technique is that it is comparatively less “fully guided” than rigid, volume-defining solutions. While the digitally planned membrane shape determines the boundary of the augmented area, the final graft volume remains dependent on intraoperative factors. Despite this limitation, the volumetric analysis demonstrated a high level of agreement between the planned augmentation volume and the achieved hard tissue gain at 9 months (Spearman’s ρ = 0.825, *p* = 0.0004). These findings suggest that the digital planning of the membrane configuration can still provide a reliable spatial framework for augmentation. In the aforementioned study by Soares et al. [35], an overall graft stability of approximately 83% was reported. Although volumetric stability could not be assessed in the present study, the strong correlation observed suggests a similarly consistent relationship between planned and achieved augmentation volumes, albeit measured using a different metric.

At certain measurements sites the horizontal dimension of the edentulous ridge 1 mm apical to the alveolar crest showed a reduction between the baseline and the 9-month follow-up CBCT scans. This finding may be explained by the tent-pole augmentation approach, as the membrane is folded over the relatively narrow screw heads, the contour of the augmented ridge may become constricted coronally, contributing to a reduction in ridge width at the crestal level. In future cases tenting screws with screw heads (e.g. SCHLEE Umbrella Screw, Ustomed Instrumente GmbH, Tuttlingen, Germany) might be utilized to overcome this limitation.

Several limitations of this study must be acknowledged. First, the volumetric comparison did not account for potential differences in the spatial characteristics of the actual augmented tissue compared to the preoperative planning. Although the total volumes were similar, variations in graft shape or positional distribution may have occurred. In addition, the digital planning workflow is most effective with membranes that maintain their predefined geometry. Cross-linked collagen membranes may stretch or deform after hydration, making them less suitable for this approach. Furthermore, the present study primarily focused on the radiographic evaluation, thus histological evaluation was not performed. The retrospective study design and the relatively small sample size may further limit the generalizability of the findings and weaken the conclusions. Moreover, the absence of a parallel control group treated with conventionally planned and performed GBR procedures precludes direct comparative analysis. Finally, patient-related outcomes were not assessed; therefore, the potential clinical benefits of the workflow, such as reduced surgical time, could not be evaluated. As a result, it is not possible to definitively determine whether the observed clinical and radiographic outcomes are superior, equivalent, or inferior to those achieved using traditional planning approaches. Future prospective, randomized controlled trials with larger cohorts are therefore necessary to validate these preliminary findings and to enable robust comparison with established GBR protocols.

Within these limitations, the present findings suggest that digitally driven, reverse-planned GBR using PTFE membranes can be a viable and accessible approach for the reconstruction of advanced alveolar ridge defects. Form-stable materials, such as the currently utilized dense PTFE (permamem^®^, botiss biomaterials GmbH, Zossen, Germany) appear well suited for this application. Overall, the workflow can be readily interpreted in contemporary clinical environments and could serve as an intermediate solution between conventional GBR and fully customized systems (CTMs and CABBs). However, large-scale comparative analyses must be conducted to further evaluate their efficacy, including patient-centered outcomes.

## Supplementary Information

Supplementary Material 1.

## Data Availability

Data is available from the authors upon reasonable request.
